# Premature placental senescence in early-onset preeclampsia: syncytiotrophoblast SASP and the maternal syndrome

**DOI:** 10.3389/fpubh.2026.1863387

**Published:** 2026-07-14

**Authors:** Meifang Zhou, Shuni Zhang, Shihao Hong

**Affiliations:** 1 Assisted Reproduction Unit, Department of Obstetrics and Gynecology, Sir Run Run Shaw Hospital, School of Medicine, Zhejiang University, Hangzhou, China; 2 Zhejiang Provincial Clinical Research Center for Reproductive Health and Disease, Hangzhou, China; 3 Dalian Medical University, Dalian, China

**Keywords:** early-onset preeclampsia, endothelial dysfunction, extracellular vesicles, liquid biopsy, placental senescence, SASP, syncytiotrophoblast

## Abstract

Early-onset preeclampsia (EOPE) remains a leading cause of maternal and perinatal morbidity worldwide. This review synthesizes evidence that premature placental senescence, centered on syncytiotrophoblast stress and the senescence-associated secretory phenotype (SASP), provides a mechanistic framework connecting placental injury to the maternal syndrome. In EOPE, defective spiral artery remodeling (45) and repeated ischemia–reperfusion can promote oxidative damage, persistent DNA-damage responses, impaired autophagy, and durable cell-cycle arrest in trophoblast lineages. Convergent data show elevated senescence markers (p16INK4a, p21, SA-*β*-galactosidase, *γ*-H2AX), transcriptomic signatures consistent with placental SASP programs, and senescent syncytiotrophoblast secretion—PAI-1 (~4-fold) and activin A (~2-fold). The SASP can transform the placenta into a source of cytokines, antiangiogenic factors, cell-free nucleic acids, and extracellular vesicles that may bias the maternal circulation toward endothelial dysfunction. We propose a stage-based model of EOPE progression and discuss differences from late-onset disease. Senescence-linked circulating signals may support multimarker risk stratification and liquid-biopsy strategies. We emphasize that human causal evidence remains associative and the senescence model is best regarded as a testable framework complementing established immune, complement, and angiogenic models.

## 1 Introduction

Preeclampsia (PE) affects 2%–8% of pregnancies worldwide and accounts for an estimated 50,000–60,000 maternal deaths annually—over 99% in low- and middle-income countries. It is simultaneously a placental disease, a maternal vascular syndrome, and a heterogeneous clinical entity, associated with substantial maternal morbidity, indicated preterm delivery (~15%–20% of indicated preterm births), fetal growth restriction (complicating 30%–50% of EOPE cases), and perinatal loss (1). The same diagnostic label covers biologically distinct routes to disease, especially when early-onset (EOPE, <34 weeks) and late-onset (LOPE, ≥34 weeks) cases are considered together (2).

The traditional two-stage model retains explanatory power: placental maldevelopment comes first (defective spiral artery remodeling detectable as early as 8–12 weeks), and the maternal syndrome follows when placental stress signals exceed maternal adaptive capacity (2). But this model is now best viewed as a scaffold rather than a complete mechanism—it tells us when the disease unfolds, not how the diseased placenta is transformed into a systemic maternal disorder. Recent work, including the updated “preeclampsia 2.0” discussion, emphasizes that the bridge between those stages must be specified more precisely (3, 4).

One of the most useful advances has been the identification of syncytiotrophoblast stress as that bridge, most comprehensively articulated by Redman et al. (3). Their framework established that stressed syncytiotrophoblast releases factors driving the maternal syndrome, providing the conceptual foundation on which a senescence-centered interpretation can be built (5, 6).

We argue that, in EOPE, syncytiotrophoblast stress can be interpreted productively through the framework of premature placental senescence (5–7). This shift matters because it moves the discussion away from a checklist of abnormal molecules and toward a coherent stress-failure program: oxidative damage is not adequately cleared, autophagic rescue is insufficient, cell-cycle arrest pathways are engaged, and the stressed trophoblast begins to reshape its environment through secretory signaling (8, 9).

That distinction is important because senescence is not a passive endpoint: a senescent trophoblast is metabolically active, secretory, and biologically influential (6, 7). Through the SASP, it can alter neighboring cells, polarize immune cells, amplify angiogenic imbalance, and expose the maternal endothelium to more toxic placental output (9–14).

The evidentiary base has strengthened considerably. Scaife et al. (8) reported elevated p16INK4a and p21 immunostaining with oxidative stress markers (8-OHdG) in preeclamptic versus control placentas. Cindrova-Davies et al. (11) documented senescence marker trajectories across gestational ages in normal and pathological placentas (detailed in Section 3). Transcriptomic studies have characterized senescence-associated placental programs (12). Nonn et al. (6) showed senescent syncytiotrophoblast secretion—PAI-1 (~4-fold) and activin A (~2-fold)—is detectable in EOPE, while Roh et al. (7) demonstrated shared placental senescence markers between peripartum cardiomyopathy and preeclampsia. These data support treating placental senescence as a serious mechanistic hypothesis, while acknowledging most studies remain cross-sectional and associative (15, 16).

Accordingly, this review examines EOPE through a narrower chain of events—maladaptive placentation, syncytiotrophoblast stress, premature placental senescence, SASP-mediated signal export, and maternal endothelial injury (3, 5, 17)—while acknowledging complementary frameworks including decidual NK cell-mediated immune dysregulation, complement activation, and the sFlt-1/PlGF angiogenic axis (18–23). The central question shifts from whether placental stress exists to how that stress is stabilized, exported, and converted into systemic disease (4, 6, 16).

A senescence-centered approach gives the transition from placental injury to maternal disease a recognizable biological direction. Rather than viewing the placenta as a passive site releasing a changing mixture of harmful factors, this model treats it as an organ entering a relatively stable stress program: oxidative stress, angiogenic imbalance, vesicle shedding, and inflammatory signaling are not competing explanations but different expressions of a placenta that has lost equilibrium and begun exporting that disequilibrium into the maternal circulation.

## 2 Scope and rationale of this narrative review

This narrative review focuses on EOPE as the placental-predominant end of the preeclampsia spectrum. Rather than attempting to catalog every pathway implicated in PE, it selectively synthesizes literature that helps explain how abnormal placentation is translated into a maternal vascular syndrome through syncytiotrophoblast stress and senescence-linked signal export. Particular attention is given to studies of human placental tissue and trophoblast stress biology (3, 6), placental transcriptomics (12, 24), extracellular-vesicle biology (13, 14), and emerging liquid-biopsy approaches relevant to EOPE (25).

The review is intentionally interpretive rather than exhaustive. The goal is not to claim senescence fully explains every form of preeclampsia—a position inconsistent with clinical heterogeneity—but to examine whether it provides a tighter organizing framework for EOPE than the diffuse language of inflammation alone (5, 6). Selection of primary literature emphasizes studies that directly characterize placental senescence markers, SASP components, or syncytiotrophoblast stress signatures in EOPE, with preference for work providing quantitative comparisons against gestational-age-matched controls.

A narrative review is useful because the relevant literature has expanded across several partially overlapping domains—placental pathology, trophoblast cell biology, senescence research, EV biology, and clinical biomarker studies—that are rarely synthesized within a single framework, and a formal systematic review with meta-analysis would be unlikely to resolve mechanistic questions when the primary studies use different markers, gestational ages, and outcome definitions. Where possible, quantitative estimates are cited directly from the source literature rather than paraphrased qualitatively.

The framework adopted here is intentionally conservative. We do not assume every marker linked to oxidative stress or cell-cycle arrest proves a fully developed senescence program, and we distinguish physiological aging, adaptive stress, and pathological senescence to avoid the claim that any senescent-like signal in a preeclamptic placenta is automatically disease-relevant (5, 11).

## 3 Literature search methods

This narrative review employed a structured literature search to enhance transparency and reproducibility. Electronic searches were conducted in PubMed/MEDLINE, Web of Science, and Scopus for articles published between January 2010 and March 2026. The primary search strategy combined the following terms: (“preeclampsia” OR “early-onset preeclampsia”) AND (“placental senescence” OR “cellular senescence” OR “syncytiotrophoblast” OR “SASP” OR “senescence-associated secretory phenotype”) AND (“extracellular vesicles” OR “sFlt-1” OR “PlGF” OR “biomarkers” OR “autophagy” OR “oxidative stress”).

Additional targeted searches were performed for specific subtopics including decidual NK cell biology, complement activation in preeclampsia, spiral artery remodeling, and senotherapeutic strategies. Reference lists of key articles and relevant reviews were hand-searched to identify additional publications. Inclusion criteria prioritized: (i) original human placental studies with clearly defined preeclampsia diagnostic criteria, (ii) studies that distinguished EOPE from LOPE where possible, (iii) transcriptomic or proteomic analyses of placental senescence, and (iv) translational studies of circulating biomarkers linked to trophoblast biology.

Studies were excluded if they lacked gestational-age-specific data, did not distinguish preeclampsia from other hypertensive disorders of pregnancy, or reported only *in vitro* findings without human tissue validation. Given the narrative nature of this review, no formal quality scoring or meta-analytic synthesis was performed; articles were selected based on their relevance to the conceptual framework and the strength of evidence they provided. The search was last updated on March 15, 2026.

## 4 Physiological senescence control and syncytiotrophoblast homeostasis in normal pregnancy

Placental senescence cannot be interpreted in a purely pathological sense without reference to normal trophoblast biology. In a healthy pregnancy, the villous trophoblast already operates close to a physiological stress threshold because it must sustain continuous turnover, syncytial fusion, endocrine activity, and barrier integrity across gestation (3, 5, 11). The key issue is therefore not whether senescence-related features can be detected in placenta, but when, where, and with what functional consequences they emerge.

The placenta is not a static organ. Even in uncomplicated pregnancy, villous tissue is characterized by high metabolic activity, continuous remodeling, and progressive accumulation of oxidative modifications (5). The syncytiotrophoblast operates close to a physiological ceiling: it must process high oxygen fluxes while maintaining barrier integrity, endocrine function, and immunological tolerance. Pathways central to senescence—DNA-damage responses, cell-cycle regulation, and redox-sensitive signaling—are already engaged in normal placental physiology, and the distinction between adaptation and pathological decline depends on timing and degree rather than on a single molecular switch (9, 11).

### 4.1 Cellular turnover in the villous trophoblast

The villous trophoblast is maintained by continuous input from proliferative cytotrophoblasts and by the differentiated syncytiotrophoblast covering the villous surface (3). This turnover system balances fusion, repair, shedding, endocrine output, transport, and barrier integrity throughout gestation (5). In quantitative terms, the syncytiotrophoblast releases approximately 1–3 g of syncytial material daily into the maternal circulation—including extracellular vesicles, syncytial knots, and cell-free nucleic acids—demonstrating that even physiological turnover involves substantial maternal-facing export. Cindrova-Davies et al. (11) demonstrated that senescence markers—SA-*β*-galactosidase activity, p16INK4a expression, telomere length reduction, and *γ*-H2AX foci—increase progressively from preterm to term in uncomplicated pregnancies, establishing a quantitative baseline for physiological placental aging.

### 4.2 Physiological aging versus pathological premature senescence

Term placentas can show senescence-like features without being pathologic (5). Premature senescence differs from this physiological aging trajectory: it appears too early, is coupled to stress accumulation, and is associated with functional decline rather than orderly maturation (8, 9). The marker trajectories that define the normal baseline are presented in Section 3 (Cellular Turnover in the Villous Trophoblast); keeping physiological aging and pathological senescence separate is essential to avoid misreading term changes as disease (11).

The syncytiotrophoblast makes this distinction challenging because differentiation and stress adaptation can resemble senescence (5, 9). The operational criteria proposed in Section 5 (Premature Placental Senescence in Early-Onset Preeclampsia) provide a single canonical working framework for diagnosing pathological premature senescence, encompassing both positive diagnostic criteria and falsification conditions. No single marker—p16INK4a, p21, SA-*β*-Gal, *γ*-H2AX, or lamin B1 loss—is sufficient in isolation (11).

### 4.3 Why syncytiotrophoblast stress matters in EOPE

The syncytiotrophoblast deserves central attention in EOPE because it is the primary interface through which placental distress reaches the mother (3). It determines whether fluctuations in perfusion and redox burden remain transient physiological challenges or are reformulated into signals with systemic reach (3, 17). A senescence-aware reading helps explain why purely descriptive references to placental inflammation are often incomplete as mechanistic explanations: inflammation may be a consequence of a prior change in secretory state rather than its primary cause (5, 6).

The syncytiotrophoblast performs a selective interpretive function: it serves as both a sensor of local conditions and a director of maternal exposure, determining which stress signals are contained and which are exported (3, 17). This places it at the center of disease progression in EOPE—governing whether placental injury remains locally buffered or is reformulated into signals with systemic reach (5, 6, 13).

For the senescence model to be most useful, it should explain not only that the placenta is stressed but through which cellular states and export mechanisms that stress reaches the mother (13, 16).

### 4.4 Senescence, placental perfusion, and barrier function

A senescent syncytiotrophoblast is more than an injured surface (5)—it is a functional liability. Once stress adaptation fails, endocrine precision, transport efficiency, redox buffering, and barrier integrity deteriorate concurrently. Barrier failure should be understood broadly: the syncytiotrophoblast is a biochemical and signaling barrier as much as a structural one, and its failure to contain and process stress signals allows locally generated mediators to access the maternal compartment. That is why even partial syncytial barrier compromise can have systemic consequences out of proportion to the anatomical scale of the lesion (3, 17), and why biomarker signatures may emerge before histopathological changes are prominent (13, 24).

## 5 Premature placental senescence in early-onset preeclampsia

We use the term premature placental senescence to describe a state in which placental injury is no longer effectively resolved, secretory signaling becomes persistently altered, and the placenta behaves as a source of systemic stress transmission rather than a buffered exchange organ (5, 6, 9). In operational terms, this transition is suggested when: (a) at least two senescence markers (e.g., p16INK4a and/or p21 upregulation, together with SA-*β*-Gal activity or *γ*-H2AX foci) are detected in villous trophoblast before 34 weeks; (b) sustained DNA-damage response signaling is evident; (c) SASP factor secretion is altered (e.g., elevated IL-6, IL-8, PAI-1, activin A relative to gestational-age-matched controls); and (d) trophoblast renewal or barrier integrity is functionally impaired. Conversely, the senescence interpretation is challenged when stress markers are present but secretory reprogramming and cell-cycle arrest are absent, or when markers resolve after removal of the presumed insult.

Premature placental senescence should therefore be understood as a process rather than a label. The relevant question is not whether a diseased placenta is simply “senescent” in a binary sense, but whether cumulative injury has pushed trophoblast biology toward durable cell-cycle restraint, maladaptive secretory activity, and impaired structural renewal. This process-based view is especially important in placental research, where differentiation, maturation, and stress adaptation can partially mimic one another. The convergence of timing, pathology severity, and molecular stress signatures strengthens the interpretation in EOPE (6, 7, 12).

A senescence-centered model connects classical maldevelopment and malperfusion theories with more recent work on placental secretome changes (3, 4). It proposes that persistent placental stress is converted into a stable secretory phenotype with inflammatory and antiangiogenic consequences. Whether sFlt-1 is part of the syncytiotrophoblast SASP or a parallel pathway remains unresolved (20, 21, 26). Current evidence favors a model in which sFlt-1 excess and placental senescence are mechanistically adjacent: both can be driven by shared upstream insults and converge on maternal endothelial dysfunction, but each may operate independently (21, 26).

### 5.1 From syncytiotrophoblast stress to a senescent placenta

In EOPE, the maternal syndrome is preceded by a longer phase of placental injury (3, 5, 17). Defective trophoblast invasion and incomplete spiral artery remodeling (27) establish a state of chronic malperfusion, repeated ischemia–reperfusion, and cumulative oxidative stress (6, 8, 9, 27, 28). The transition from adaptive stress to premature senescence is unlikely to occur as a single event. More plausibly, trophoblasts pass through increasingly durable stress states in which cell-cycle arrest, oxidative injury, and secretory alterations progressively deepen (5, 12, 14). At the inflection point where SASP becomes sustained and trophoblast quality control fails, local placental stress crosses into a propagating maternal signal (13, 16, 25, 29).

During early phases of stress, oxidative and metabolic disruption may be partly buffered through mitochondrial adaptation and autophagic clearance. As protective systems lose efficiency—potentially weeks before clinical diagnosis—damage signaling becomes more durable and secretory reprogramming stabilizes. By the time delivery specimens are examined, the placenta may contain the accumulated imprint of a prolonged failure of recovery rather than a simple snapshot of terminal injury. This temporal uncertainty is a fundamental limitation of delivery-only study designs (5, 9, 16).

### 5.2 Why EOPE is the most informative clinical context

EOPE is the clearest setting to examine this model because the placenta is usually the dominant driver (2, 6). Histologic injury is more substantial, angiogenic imbalance is more marked (sFlt-1/PlGF ratios often exceed 100 versus <50 in most LOPE), and perinatal outcomes are worse than in LOPE (2, 16). When disease begins early and placental pathology is prominent, there is less ambiguity about whether senescence-associated changes are likely to be causal participants rather than secondary phenomena (6, 16).

That clinical context is important methodologically as well as biologically. When disease begins early and placental pathology is prominent, there is less ambiguity about whether senescence-associated changes are likely to be causal participants. By contrast, in milder disease or late presentations that overlap with normal term changes, the signal-to-noise ratio deteriorates (2, 5, 16).

### 5.3 SASP as an amplifier of sterile inflammation

Sterile inflammation—inflammation occurring in the absence of detectable pathogens, driven by endogenous damage-associated molecular patterns (DAMPs) released from stressed or dying cells—is a hallmark of the SASP-driven amplification phase. The pathogenic force of senescence in EOPE lies less in growth arrest itself than in what senescent cells start to release (6, 7). Once the trophoblast enters a SASP state, it generates a local environment enriched in inflammatory cytokines, alarmins, proteases, extracellular-vesicle cargo, and antiangiogenic signals (13, 14, 25, 30). This turns a stressed placenta into an active amplifier of sterile inflammation (10, 24).

The value of invoking SASP is that it shifts discussion from isolated cytokine elevations toward coordinated secretory behavior that can alter neighboring trophoblast behavior, reshape immune-cell recruitment, and reinforce oxidative and angiogenic stress within villous tissue—helping explain persistence, amplification, and feed-forward signaling rather than one-time injury alone.

### 5.4 Spillover into the maternal circulation

After that transition, SASP-related mediators and syncytiotrophoblast-derived extracellular vesicles reach the maternal circulation (13, 14), where antiangiogenic factors and nucleic acids may further injure endothelial cells, disturb vascular tone, and intensify complement and inflammatory pathways (15, 16, 25, 29).

Once placental output reaches the maternal circulation, the biological importance of packaging becomes central. Soluble mediators, EVs, and nucleic acids do not circulate as interchangeable signals; their packaging influences stability, tissue targeting, and biological meaning. A placenta that packages stress signals into stable carriers with endothelial tropism may generate maternal consequences quite different from one releasing similar mediators without such protection (13, 31, 32).

## 6 Relationship to alternative mechanistic frameworks

A senescence-centered interpretation of EOPE gains credibility by demonstrating how alternative models relate within a broader mechanistic landscape. Several frameworks deserve explicit attention because they address processes that the senescence model alone does not fully explain.

### 6.1 Decidual NK cells and spiral artery remodeling

The decidual immune interface, particularly maternal dNK cell interactions with fetal extravillous trophoblast, represents a thoroughly characterized alternative framework (22, 23). dNK cells are the dominant leukocyte population at the implantation site and regulate spiral artery remodeling through cytokines, angiogenic factors, and matrix metalloproteinases. Hiby et al. (22) demonstrated that maternal KIR AA genotype combined with fetal HLA-C2 alleles increases preeclampsia risk, suggesting inadequate dNK activation impairs trophoblast invasion; Moffett and Colucci (23) mechanistically reviewed this system. The senescence and dNK models address different temporal windows: the dNK framework explains why placentation may be deficient, while the senescence framework explains how that deficiency is converted, via chronic stress, into a systemic maternal syndrome.

### 6.2 The sFlt-1/PlGF angiogenic axis

The sFlt-1/PlGF pathway is the most clinically validated mechanistic axis in preeclampsia, supported by evidence that excess placental sFlt-1 causes hypertension and proteinuria in animal models (20), that circulating sFlt-1 rises and PlGF falls weeks before clinical onset (21), and that the sFlt-1/PlGF ratio can rule out preeclampsia with high negative predictive value (26). Whether sFlt-1 is part of the SASP or a parallel pathway remains unresolved. For translational purposes, angiogenic biomarkers and senescence-linked signals likely provide complementary rather than redundant information about placental status (24).

### 6.3 Complement activation

Complement dysregulation is well documented in preeclamptic placentas and maternal circulation, with elevated plasma C5a (2- to 3-fold), C3a, and soluble C5b-9 in established preeclampsia (14, 32, 33). Complement may be triggered by syncytiotrophoblast debris, EVs, or DAMPs released from stressed trophoblasts, creating a potential link to senescence-associated secretion. However, complement can also be activated independently through classical, lectin, or alternative pathways without a senescence trigger. Studies directly testing whether SASP factors activate complement, or whether complement effectors reinforce trophoblast senescence, are largely absent and represent a research priority.

### 6.4 Maternal constitutional factors in LOPE

In late-onset disease, maternal cardiometabolic predisposition—including obesity (BMI ≥ 30 kg/m^2^, ~30–40% of LOPE cases), insulin resistance, chronic hypertension, and dyslipidemia—may contribute to placental stress independently of primary placental maldevelopment (2, 34–36). Uterine artery Doppler indices are often normal or only mildly elevated in LOPE, contrasting with the markedly abnormal waveforms typical of EOPE (2). Senescence-like features may represent an exhausted placental response to maternal metabolic burden rather than a placenta-initiated process, with practical implications: biomarkers developed in EOPE-enriched cohorts may perform poorly in LOPE populations, and aspirin prophylaxis shows limited efficacy against term disease (37). The senescence model posits that the same placental stress-response programs can be engaged by different upstream drivers with differing implications for timing, severity, and reversibility.

## 7 Major cellular manifestations of a senescent placental phenotype

A senescence-centered interpretation is most convincing when mapped onto specific placental cell populations. The following sections examine syncytiotrophoblast, cytotrophoblast, Hofbauer cells, and the maternal-fetal interface as sites where senescence-associated changes carry distinct functional consequences.

At the tissue level, a senescent placental phenotype reflects a disturbed multicellular ecosystem in which the syncytiotrophoblast is the dominant source of exported signals while underlying cytotrophoblast and stromal populations influence whether senescence can be reversed or contained (6, 7).

### 7.1 Syncytiotrophoblast as the primary site of SASP generation

Among placental compartments, the syncytiotrophoblast is the most plausible origin of clinically consequential signal export because it directly interfaces with maternal blood. Even modest changes in its secretory state can have systemic effects disproportionate to the anatomical scale of the lesion, and signals released from this layer bypass stromal buffering to reach the maternal vasculature with minimal dilution (3, 6, 13, 24).

#### 7.1.1 Physiological role of the syncytiotrophoblast

In normal pregnancy, the syncytiotrophoblast is best understood as an adaptive, highly specialized interface (3). It must sense oxygen and nutrient conditions, coordinate endocrine signaling, and preserve barrier function while undergoing constant turnover (5). Under physiological conditions, this layer contains stress without converting it into inflammatory spillover (6).

#### 7.1.2 Premature syncytial senescence in EOPE

Recent studies bring greater precision to the claim that the syncytiotrophoblast may adopt senescence-like states in EOPE (6, 7). The strongest evidence is no longer indirect staining alone; it now includes direct characterization of senescent syncytiotrophoblast secretion, alongside transcriptomic data showing placental senescence-associated programs (12). These observations make the syncytiotrophoblast a plausible major site of SASP generation in placental-dominant PE, while still requiring careful interpretation of timing and cellular context (15).

Even here, caution is required. The strongest studies do not establish that every syncytiotrophoblast in EOPE is uniformly senescent, nor do they imply that a single marker defines that state across patients. They suggest that a proportion of syncytiotrophoblast adopts senescence-like features in EOPE and that this proportion may be large enough to alter the aggregate release of biologically active signals into the intervillous space (6, 12, 18). Which patients show the most pronounced senescence, and at what gestational point it first becomes detectable, remains to be determined.

### 7.2 Cytotrophoblast exhaustion and impaired renewal

Senescence biology in EOPE should not be reduced to changes in the mature syncytium alone. Because villous homeostasis depends on continued recruitment and differentiation of competent cytotrophoblasts, disruption of the regenerative pool can amplify syncytial fragility and make the placenta less capable of recovering from recurrent malperfusion stress (8, 9, 11).

Because villous homeostasis depends on continued recruitment and differentiation of competent cytotrophoblasts, senescence biology in EOPE cannot be reduced to changes in the mature syncytium alone. If the cytotrophoblast pool becomes stressed, exhausted, or depleted, the syncytium loses regenerative reserve (8–10).

#### 7.2.1 Normal proliferative support

Syncytial integrity depends on continued recruitment of competent cytotrophoblasts. In a healthy placenta, that renewal circuit keeps fusion, repair, and shedding in balance. If the cytotrophoblast pool becomes stressed or exhausted, the syncytium is left with less regenerative reserve and more pressure to function while damaged (11).

#### 7.2.2 Impaired replenishment in senescent placentas

Chronic placental stress matters even before the maternal syndrome becomes obvious (8, 10). Oxidative stress, defective autophagic clearance, and persistent DNA-damage signaling do not affect the syncytium in isolation; they also progressively deplete or compromise the cytotrophoblast pool. When regeneration cannot keep pace with syncytial damage, a self-reinforcing dynamic emerges in which declining repair capacity increases stress on the remaining syncytium and further reduces regenerative input (9, 30).

#### 7.2.3 Consequences for villous integrity and shedding

At the tissue level, this failure of renewal may appear as syncytial instability, abnormal shedding, altered nuclear clustering, and increased release of syncytial debris and vesicles (3). These features are not trivial by-products (6). They provide plausible vehicles by which a senescent placenta broadcasts distress to the maternal circulation (13).

### 7.3 Hofbauer cells and the senescence-inflammation circuit

Immune remodeling becomes mechanistically important when placental senescence is viewed as a self-reinforcing process rather than a static histological label. In that setting, macrophage populations are relevant not simply because they are present in injured villi, but because they may determine whether stress signals are cleared, contained, or amplified into a sustained inflammatory circuit (28, 34, 38).

The inclusion of placental macrophages within this model is important because it widens the framework from intracellular stress to microenvironmental reinforcement. Once senescent trophoblasts begin secreting SASP factors, macrophages responding to those signals may themselves become sources of additional inflammatory mediators, creating a self-reinforcing loop in which senescence and inflammation are no longer separable processes (29, 35). This is not a claim that every macrophage response is senescence-driven, but that SASP-associated signals can bias placental immune function toward chronic inflammation (29).

#### 7.3.1 Homeostatic macrophage functions

Hofbauer cells and related placental macrophage populations normally help maintain villous homeostasis through debris clearance, remodeling support, and calibrated inflammatory control (38). In a healthy placenta, those functions limit the escalation of tissue injury (34).

#### 7.3.2 Pro-inflammatory coupling with SASP

A trophoblast SASP can disrupt that balance (25). Alarmins such as HMGB1 and other senescence-linked mediators can recruit monocytes, bias macrophage polarization, and create a feedback loop in which stress and inflammation sustain one another (28, 38). This does not mean macrophages start the disease; rather, they become secondary amplifiers once the senescent program is established.

#### 7.3.3 Parenchymal cross-talk and immune remodeling

The result is a placental microenvironment in which senescence and inflammation are no longer separable processes (28, 38). Senescent trophoblasts alter immune-cell behavior, while inflammatory cells reinforce trophoblast dysfunction. That reciprocity helps explain why isolated measurement of inflammatory cytokines often feels descriptive, whereas a senescence-centered interpretation is mechanistically tighter (4, 34, 35).

#### 7.3.4 Endothelial-oriented signal amplification

These signals matter systemically when they are repackaged into stable carriers or combined with classic endothelial toxins (13, 24). Extracellular vesicles, sFlt-1, complement-associated cargo, and related factors extend the biological reach of placental injury and give SASP-associated signals access to maternal vascular targets (14, 32).

### 7.4 Attenuation of reparative signaling at the maternal-fetal Interface

A further consequence of premature placental senescence is the loss of reparative flexibility (5, 6). Once a trophoblast population becomes locked into persistent stress signaling and inadequate clearance, the interface is less able to return to homeostasis even if the original insult fluctuates (10). In practice, this means the placenta becomes easier to destabilize and harder to rescue (9).

## 8 Key molecular pathways

No single pathway is sufficient to define pathological placental senescence, but several signaling domains recur across the current literature: oxidative injury, DNA-damage responses, cell-cycle arrest, SASP composition, extracellular vesicle biology, and autophagic failure. Organizing the literature around these pathways prevents mechanistic fragmentation—oxidative injury, mitochondrial dysfunction, defective autophagy, DNA-damage signaling, and SASP regulation are interacting components of a broader trophoblast stress network in which each influences the others’ progression (8–10).

These pathways should be interpreted as a network rather than a checklist. A useful mechanistic reading asks not which pathway is singly decisive, but how their interaction creates a threshold beyond which recovery becomes progressively less likely. In EOPE, repeated ischemia–reperfusion may produce exactly the conditions under which multiple stress pathways reinforce rather than compensate for one another (8–10).

### 8.1 Oxidative stress, mitochondrial dysfunction, and DNA-damage response

Among the proposed upstream drivers, oxidative stress remains the best established (8, 17). Intermittent perfusion, mitochondrial dysfunction, lipid peroxidation, and redox-sensitive signaling together create a setting in which trophoblast injury is repeatedly generated faster than it can be resolved (10, 29). Several lines of evidence now link this redox burden to premature placental senescence rather than to nonspecific damage alone (9, 28, 32).

These processes are especially relevant because they link a hemodynamic abnormality to a cell-state abnormality. Disturbed perfusion is not pathogenic simply because oxygen delivery fluctuates, but because repeated metabolic instability alters mitochondrial performance, increases redox burden, damages macromolecules, and eventually engages nuclear stress responses that are difficult to reverse. In this way, oxidative stress and mitochondrial dysfunction act not merely as correlates of placental injury but as major upstream conditions under which senescence becomes biologically plausible.

### 8.2 Cell-cycle arrest pathways: p53/p21 and p16INK4a/Rb

The core molecular signature of senescence is sustained cell-cycle withdrawal, commonly organized through p53/p21 and p16INK4a/Rb-associated pathways (6, 7). In placenta, however, these pathways should not be interpreted simplistically as proof of one homogeneous state (8, 10). Their expression can overlap with differentiation and adaptive stress responses. Even so, when cell-cycle arrest markers appear alongside oxidative stress, secretory activation, and functional injury—particularly in preterm EOPE placentas—they provide stronger support for a pathological senescence program (9, 32).

Interpretation of these pathways in placental tissue requires caution because differentiation, quiescence, and senescence can share partially overlapping molecular signatures. Even so, persistent engagement of p53/p21 and p16INK4a/Rb-associated programs gains stronger mechanistic significance when it is coupled to evidence of unresolved oxidative stress, dysfunctional organelle clearance, and altered secretory behavior. In that setting, cell-cycle arrest is less likely to represent a transient protective pause and more likely to indicate that trophoblasts have entered a stabilized non-proliferative stress state.

### 8.3 SASP composition: cytokines, activin A, and proteases

The placental SASP is unlikely to be static (6, 7); its composition probably varies with cell type, gestational age, and initiating insult (11, 12). In canonical senescence biology, the SASP typically includes pro-inflammatory cytokines (IL-6, IL-8), chemokines, matrix metalloproteinases, and growth factors, regulated by NF-κB, p38MAPK, and DNA-damage response signaling (39). Current placental data point to analogous components—inflammatory cytokines and proteases, alarmins and activin-related signals, extracellular-vesicle cargo, and angiogenic regulators (13, 14, 24, 25, 29, 30)—shifted in disease-specific ways (12).

Heterogeneity in SASP composition is the expected consequence of a context-dependent tissue response across multiple trophoblast states, gestational ages, and stress modalities. What matters mechanistically is that senescence is associated with a coordinated shift toward signals that propagate injury, remodel neighboring cells, and extend placental stress into the maternal compartment.

### 8.4 Extracellular vesicles, cfDNA, and senescence signal export

One reason this model is attractive is that placental senescence is hypothesized to leave circulating traces (13, 24). Whether EVs, cfDNA, and methylation-derived signals faithfully preserve aspects of trophoblast biology after release into maternal blood remains an area of active investigation; direct experimental evidence that senescent syncytiotrophoblasts alter vesicle cargo in ways that causally affect endothelial function is currently limited. Nevertheless, the concept creates a plausible mechanistic link between placental pathology and liquid-biopsy biomarkers (14) and provides a rational translational path (25).

The maternal circulation may contain readable traces of placental cell state well before delivery, making the export perspective attractive for translation. Even if no single circulating analyte can be taken as a definitive proxy for placental senescence, patterns of vesicle cargo, nucleic-acid release, and methylation or fragmentation signatures may collectively capture whether trophoblast stress has become chronic and maladaptive. Such an approach is conceptually more faithful to the biology than searching for one universal molecule.

Autophagy and mitophagy are the major candidate pathways for stress resolution in trophoblasts (10). When they work, damaged proteins and organelles can be cleared before injury becomes fixed. When they fail, oxidative damage is retained, mitochondrial dysfunction accumulates, and senescence becomes easier to stabilize (9). The advanced oxidative protein products (AOPP)-autophagy work is particularly useful here because it provides an experimentally tractable route from oxidative burden to trophoblast senescence (30).

**Figure 1:**
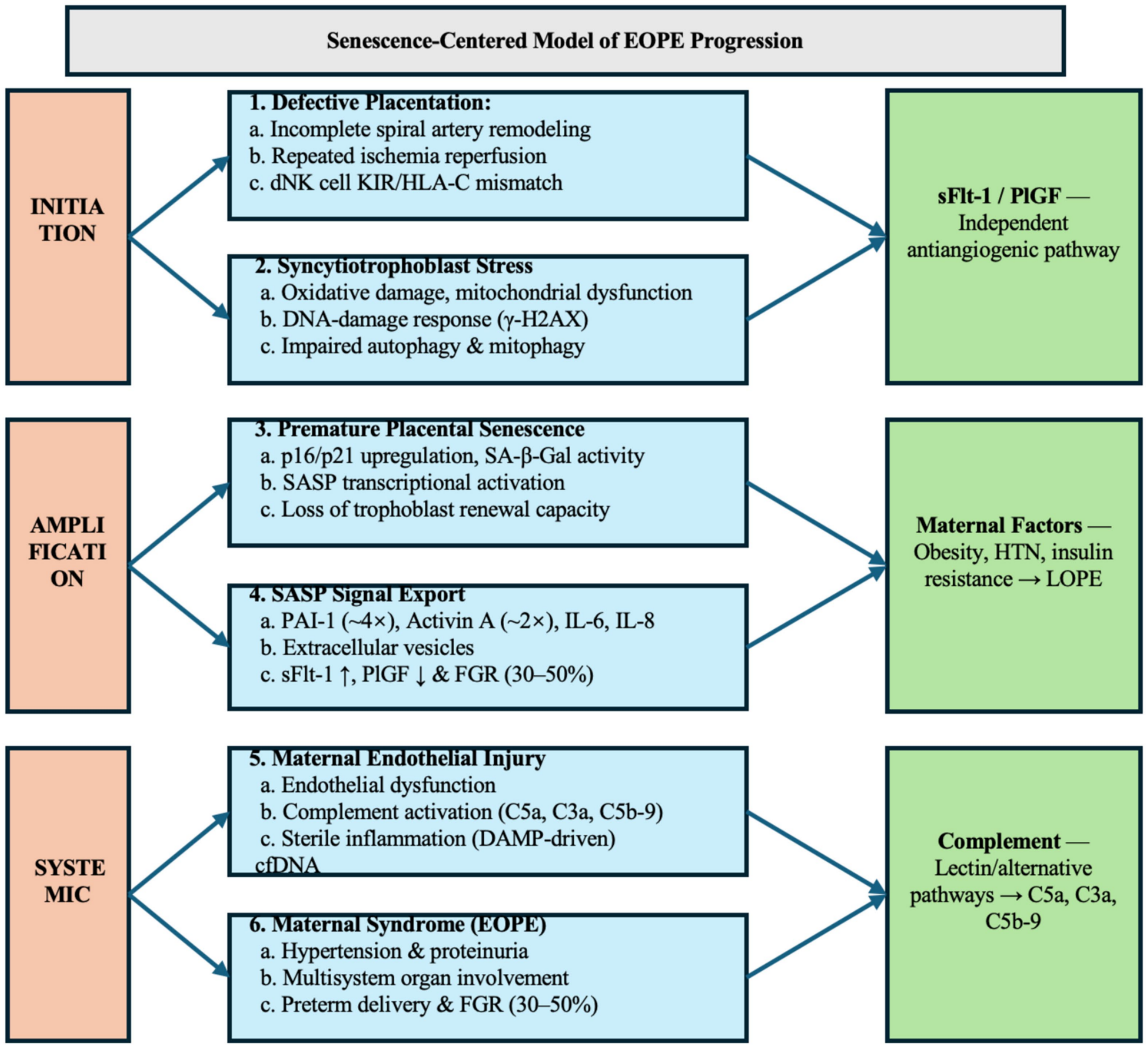
Senescence-centered model of EOPE progression. Placental stress promotes premature trophoblast senescence and SASP signaling, which contribute to endothelial dysfunction, systemic inflammation, and the development of EOPE.

Autophagic competence is especially important because it sits at the boundary between injury and adaptation. A trophoblast cell can tolerate substantial metabolic and oxidative burden if damaged organelles and proteins are efficiently removed. Once that disposal system falters, however, injury is retained rather than neutralized, mitochondrial dysfunction deepens, and pro-senescent signaling becomes easier to stabilize. This is why defects in autophagy and mitophagy are best interpreted not as secondary curiosities, but as potential turning points in the evolution from stress to durable placental dysfunction (see Figure 1).

## 9 Stage-based interpretation of EOPE progression

The value of a stage-based model is not that it imposes rigid temporal boundaries on a biologically overlapping process. Rather, it helps distinguish the transition from early placental maladaptation, to local stress amplification, to maternal vascular expression of disease, thereby making clearer where senescence may function as a mechanistic inflection point rather than merely a late morphological correlate (3, 6, 24).

A stage-based model helps distinguish escalation from mere coexistence. Many reports describe placental hypoxia, inflammatory activation, antiangiogenic excess, and maternal endothelial injury within the same study, but a stage-based interpretation imposes temporal logic—specifying which elements are likely to appear early, which amplify the process, and which mark systemic expression—making the sequence testable rather than an undifferentiated list of pathological features (3, 6, 13) (see Table 1).

**Table 1:** Senescence-centered model of EOPE progression.

Stage	Placental event	Predominant biology	Maternal relevance
Initiation	Defective placentation and limited spiral artery remodeling	Intermittent malperfusion, syncytiotrophoblast stress, redox burden	Placental reserve begins to decline before overt maternal disease
Amplification	Stress becomes persistent rather than recoverable	Senescence-associated signaling, SASP release, immune and vesicle amplification	Placental injury acquires sustained export capacity
Systemic expression	Placental output reaches the maternal circulation at higher pathogenic intensity	Antiangiogenic factors, extracellular vesicles, cfDNA and inflammatory mediators act on endothelium	Hypertension, proteinuria, endothelial dysfunction and multisystem features become clinically visible

### 9.1 Initiation stage: maladaptive placentation and excess syncytial stress

The first stage of a senescence-centered EOPE model is not simply “poor placentation” in the abstract (3, 17). It is a placenta that enters the second trimester with inadequate vascular remodeling and limited reserve to buffer ongoing perfusion stress (5, 6). From that point onward, the syncytiotrophoblast is repeatedly challenged by injury that may initially be contained but becomes progressively harder to resolve (4).

### 9.2 Amplification stage: SASP release and sterile signal expansion

The second stage begins when adaptive stress responses are no longer sufficient (6, 13). Stress is less effectively contained; a senescence-associated program may stabilize; SASP output rises; and immune amplification follows (24, 25). Extracellular-vesicle-mediated export also increases, further broadening maternal exposure (14, 38). At that point the placenta is no longer only vulnerable—it is actively producing the molecular conditions that perpetuate EOPE (28, 30).

### 9.3 Systemic stage: endothelial injury and the maternal syndrome

The maternal syndrome emerges when placental output begins to exert broader effects on maternal vascular biology (3, 24). Endothelial activation, reduced vasodilatory reserve, increased permeability, and microvascular dysfunction then become clinically visible as hypertension, proteinuria, and multiorgan involvement (14). In this reading, maternal disease can be viewed as a downstream expression of a placenta that has shifted from stressed to persistently secretory and maladaptive (32).

## 10 How this framework differs between EOPE and LOPE

Integration of multiple biomarker layers is consistent with the broader preeclampsia screening literature, in which no isolated analyte has shown sufficient performance across all phenotypes and gestational windows (40, 41). The most informative biomarker combinations are likely to differ between EOPE, LOPE, and gestational-age-matched controls. A senescence-informed strategy would be strongest not as a stand-alone assay, but as an EOPE-enriched overlay added to established angiogenic and placental-stress frameworks, especially when extracellular-vesicle biology and trophoblast-derived signatures are interpreted together rather than in parallel silos (33, 42, 43).

LOPE may also show placental aging phenomena, but the interpretation differs (5, 34). In later disease, senescence-like findings may reflect an exhausted placenta adapting to maternal cardiovascular or metabolic vulnerability—obesity, chronic hypertension, insulin resistance—rather than a placenta-initiated syndrome (4, 35). LOPE is more than twice as likely to involve maternal obesity (OR ~2.5), with uterine artery Doppler often normal (2, 36). EOPE shows abnormal first-trimester biomarkers (low PAPP-A, low PlGF), severe histopathological lesions, and fetal growth restriction (30%–50% of EOPE vs. 10%–15% of LOPE) (2, 24). The ASPRE trial demonstrated aspirin reduced preterm preeclampsia by 62% with no effect on term disease (37), reinforcing the distinction between early placental-driven and later maternal-factor-associated disease (16).

Recent reviews suggest that intervention concepts should be aligned with syncytiotrophoblast biology, including fusion control, vesicle trafficking, redox buffering, and autophagy-dependent stress resolution, rather than framed only as generalized anti-inflammatory therapy (18, 43–45). Because the syncytiotrophoblast is the primary source of exported SASP signals, strategies that improve its resilience may offer protection disproportionate to their molecular specificity. More experimental proposals such as cell-free vesicle engineering or exosome-based repair remain exploratory and should currently be discussed as future directions rather than near-clinical options (Table 2) (19).

**Table 2:** EOPE and LOPE in a senescence-centered framework.

Dimension	EOPE	LOPE	Interpretive implication
Timing	Earlier gestational presentation	Later gestational presentation	Gestational timing affects whether placental stress is likely to be primary or secondary
Placental dominance	Usually stronger	Often less dominant or more mixed with maternal host factors	The senescence-centered model is most explanatory when placental injury is early and severe
Histopathology / angiogenic imbalance	Typically more marked	Often milder or more heterogeneous	Mixed PE cohorts may dilute a placenta-specific signal
Biomarker interpretation	More suitable for EOPE-enriched senescence or trophoblast-export signatures	Signals may be confounded by broader maternal cardiovascular or metabolic vulnerability	Subtype-aware biomarker design is preferable to universal PE markers

For that reason, we favor a spectrum model with mechanistic weighting rather than a rigid binary taxonomy (4, 5). EOPE is the phenotype in which premature placental senescence most effectively organizes the available evidence; LOPE may involve overlapping pathways, but usually with weaker placental dominance (16).

## 11 Clinical significance

A related limitation is that translational enthusiasm can outpace mechanistic resolution. Reviews of exosome biology and syncytiotrophoblast targeting highlight promising intervention logic (18, 45), while putative senotherapeutic strategies underscore how incomplete the field still is with respect to tissue specificity, gestational timing, and off-target maternal-fetal effects (19). For that reason, the most defensible near-term goal is sharper biological stratification of EOPE rather than premature therapeutic extrapolation.

The practical value of a senescence-centered framework is that it may help stratify which placental signals are likely to be informative, in whom, and at what point in disease evolution. This matters because the history of preeclampsia research has repeatedly shown that markers with biologic plausibility can perform inconsistently when tested outside the specific clinical contexts in which they are generated. A conceptually tighter model may therefore improve not only candidate selection but also cohort design and interpretation.

In our view, the most persuasive current sequence is that maladaptive placentation is proposed to create repeated syncytiotrophoblast stress (5, 6); unresolved stress may favor a premature senescence program (7, 12); senescent trophoblasts adopt a SASP and can export injury signals (13, 24); and those signals thereby reshape the maternal circulation into an endothelial syndrome (25). This framework also helps connect classic placental models with newer work on extracellular vesicles, trophoblast fusion, biomarker integration, and autophagy-linked stress handling (18, 19, 33, 40, 41, 43–45).

A senescence-centered view encourages a different logic: identify when placental stress becomes persistent, determine how that persistence changes exported signals, and intervene before maternal endothelial injury becomes self-sustaining. Even if this program remains aspirational, it gives clearer direction to translational research than broad references to placental dysfunction alone.

### 11.1 Potential of biomarkers

Syncytiotrophoblast-derived EVs and senescence-associated transcripts may report a placenta that has entered a maladaptive secretory state before the maternal syndrome is fully established (6, 12). Among SASP-linked candidates, PAI-1 and activin A deserve attention: Nonn et al. (6) identified both in the senescent syncytiotrophoblast secretome in EOPE, with PAI-1 showing ~4-fold and activin A ~ 2-fold upregulation versus controls. Both are measurable in maternal circulation—PAI-1 contributes to thrombotic tendency and endothelial dysfunction, while activin A modulates trophoblast differentiation and is elevated in preeclamptic sera (6, 12). Methylation signals, angiogenic mediators, and cfDNA-based readouts may further extend the senescence signal into maternal blood (14, 25), offering candidates that preserve placental specificity while remaining accessible for risk stratification.

A useful way to conceptualize the biomarker landscape is to distinguish near-term translational markers from exploratory senescence-linked signals. The former include the sFlt-1/PlGF ratio, which has regulatory approval and demonstrates 99.3% negative predictive value for ruling out preeclampsia within 1 week (26). The latter include EV cargo profiles, senescence-associated transcript panels (PAI-1, activin A, and other SASP components), and cfDNA methylation signatures that may capture more specific information about trophoblast state, but still require analytical standardization (coefficients of variation for EV isolation currently range from 15% to 40% across laboratories), gestational-stage calibration, and external validation in EOPE-enriched cohorts (13, 24, 25).

Even so, it would be premature to treat “placental senescence” as a single biomarker class ready for routine use (24). The more defensible direction is multimarker integration: combining placental-stress signals, angiogenic markers, and senescence-linked liquid-biopsy features while enriching analyses for biologically coherent subgroups such as EOPE (25). The main translational challenge is not merely sensitivity, but biologic specificity—distinguishing a placenta undergoing maladaptive early senescence from one showing later gestational aging, nonspecific injury, or mixed maternal-fetal stress states.

Another reason to avoid a single-marker mentality is that placental senescence is unlikely to leave one exclusive circulating signature. Different patients may express the same underlying stress state with different secretory profiles—a principle well established in oncology, where single-biomarker strategies have largely been superseded by panels (24). The more defensible direction is multimarker integration combining placental-stress signals with angiogenic and metabolic readouts in longitudinal cohorts (6, 16).

Biomarker strategy should move beyond the search for a single best marker toward biologically layered profiling. A useful panel may need to capture placental stress burden, angiogenic balance, and alterations in circulating EVs or cfDNA patterns—each reflecting a different aspect of the senescence-to-signal export sequence—with the understanding that the most informative analyte combination may differ between EOPE, LOPE, and gestational-age-matched controls (13, 24, 40, 42) (see Table 3).

**Table 3:** Candidate biomarker layers in a placental senescence model of EOPE.

Biomarker layer	Examples	What it may reflect	Current translational position
Established placental-stress / angiogenic signals	sFlt-1, PlGF, related angiogenic ratios	Placental stress burden and angiogenic imbalance	Closest to current clinical use, but not fully phenotype-specific
Trophoblast-export signals	Syncytiotrophoblast-derived extracellular vesicles and vesicle cargo	Altered placental communication and maternal-facing signal export	Promising for EOPE-enriched cohorts, still analytically heterogeneous
Nucleic-acid readouts	cfDNA quantity, fragmentation or methylation-derived features	Placental injury, cell-state changes and potentially senescence-linked release patterns	Conceptually strong, but requires external validation and gestational calibration
Integrated multimarker panels	Combined angiogenic, EV and cfDNA-type profiles	Biologically layered approximation of trophoblast stress plus export behavior	Likely more faithful to disease biology than single-marker strategies

### 11.2 Directions for targeted intervention

Therapeutic ambition may usefully extend beyond blood-pressure control toward interruption of upstream stress amplification (9, 10), including improved trophoblast stress handling, restored autophagic competence (13, 16), and selective moderation of SASP-associated pathways when safe—though these remain mechanistic prospects rather than established therapies (29, 30, 32).

Several candidate senotherapeutics warrant discussion. Ergothioneine, a naturally occurring mitochondrial-protective antioxidant, reduced oxidative stress and senescence markers in trophoblast models and has been proposed as pregnancy-compatible (18). Dasatinib combined with quercetin constitutes the most studied senolytic regimen (36); however, dasatinib crosses the placenta with a near-equivalent maternal-fetal concentration ratio and documented fetal toxicity, including hydrops fetalis and hematologic suppression (46). Rapamycin, an mTOR inhibitor with senomorphic properties, can attenuate SASP but raises immunosuppression concerns during pregnancy (36). These examples illustrate that the translational gap between *in vitro* promise and safe application in pregnancy remains substantial; the most defensible direction is not systemic senolysis but placenta-aware strategies that selectively improve trophoblast stress resilience or moderate harmful secretory outputs.

Caution is necessary, however (5). Senescence during pregnancy is not uniformly harmful, and wholesale suppression of senescence pathways could disrupt normal placental maturation or repair (9). Any future intervention will have to discriminate physiological aging from maladaptive early senescence and will likely require precise timing, subtype selection, and placenta-aware safety evaluation (16).

Therapeutic implications are best framed cautiously, recognizing that no senotherapeutic agent has entered clinical trials for preeclampsia as of 2026. Interventions may prevent stress accumulation before a senescence program stabilizes—analogous to aspirin prophylaxis in screen-identified high-risk pregnancies (37)—or blunt downstream consequences of an already established secretory phenotype. A placenta early in transition may still be biologically modifiable in ways that a late, severely dysfunctional placenta is not. Development of placenta-specific delivery systems and *ex vivo* placental perfusion models for senotherapeutic screening represent critical but underdeveloped translational infrastructure.

The therapeutic relevance of this framework lies in its emphasis on timing and mechanism. Once the maternal syndrome is clinically apparent, opportunities for placenta-directed rescue may be limited; the more realistic translational goal is to act earlier—before a senescence program becomes durably established and before SASP output reaches a threshold capable of sustaining endothelial injury independently of ongoing placental stress (9, 18, 36).

The SASP itself is incompletely resolved (12, 13). Different studies emphasize different transcripts, proteins, vesicle cargo, or lipid-oxidation products (14, 30), with reported effect sizes varying considerably—for example, IL-6 elevations range from 1.5- to 8-fold across studies. The field would benefit from standardized tissue collection protocols, consensus SASP marker panels, and minimum reporting standards (36) (see Table 4).

**Table 4:** Translational intervention directions and major cautions discussed in this review.

Intervention direction	Mechanistic target	Potential rationale	Key caution
Reduce upstream stress accumulation	Perfusion-related injury, oxidative stress, mitochondrial burden	May delay the transition from adaptive stress to stabilized senescence-like dysfunction	Effective window may be early and may differ by phenotype
Improve stress resolution	Autophagy / mitophagy and organelle quality control	Could help retain trophoblast resilience before damage becomes fixed	Human causal evidence remains limited
Modulate harmful export behavior	SASP-associated pathways, vesicle release or pathogenic cargo	Targets the maternal-facing arm of placental injury amplification	Placental signaling also serves physiological functions, so nonspecific suppression may be unsafe
Biologically stratified intervention design	EOPE-enriched, placenta-dominant subgroups	Improves alignment between mechanism, timing and expected treatment effect	Requires better subtype definition and placenta-aware safety evaluation

## 12 Current controversies and limitations in research

Important uncertainties remain (5, 6). Senescence markers overlap with differentiation and stress responses, placental samples are often collected only at delivery (9), and many studies rely on cross-sectional comparisons (12). Establishing causality requires designs beyond delivery-only sampling: longitudinal first-trimester biospecimen collection, trophoblast organoid models with controlled senescence induction, single-cell and spatial transcriptomic analyses of EOPE placentas, and inducible trophoblast-specific senescence models. Until such data become available, the senescence model should be regarded as a coherent, testable interpretive framework supported by convergent indirect evidence rather than an established causal mechanism.

The SASP itself is also incompletely resolved (12, 13). Different studies emphasize different transcripts, proteins, vesicle cargo, or lipid-oxidation products (14, 30), and reported effect sizes vary considerably—for example, IL-6 elevations in preeclamptic placental tissue range from 1.5- to 8-fold across published studies. The field would benefit from standardized tissue collection protocols, consensus SASP marker panels, and minimum reporting standards analogous to those proposed by Gorgoulis et al. (36) for cellular senescence research.

Premature placental senescence provides a mechanistically coherent framework for organizing the transition from abnormal placentation to the maternal syndrome in EOPE, supported by convergent data from histology, transcriptomics, secretome characterization, and circulating biomarker studies (6, 7, 12, 13). Alternative frameworks—decidual NK cell-mediated immune regulation, complement activation, and the sFlt-1/PlGF angiogenic axis—coexist with the senescence model (18–23). The operational definition of placental senescence requires refinement through consensus criteria, and the translational promise of senotherapeutics must be tempered by recognition that senescence serves physiological functions nonselective suppression could disrupt (18, 36, 47). The most productive way forward is to treat the senescence hypothesis as a stimulus for more precise experimental design: identifying which trophoblast populations are most consequential, defining circulating signatures that most faithfully report that transition, and determining whether interventions improving trophoblast stress resolution can alter disease trajectory.

Another important limitation is conceptual. In placental biology, senescence is sometimes invoked too loosely to describe almost any combination of oxidative stress, trophoblast damage, and increased inflammatory signaling (5), risking unfalsifiability. We have proposed operational criteria above (see Section 5), but acknowledge that these require prospective validation. The field would also benefit from consensus guidelines analogous to those established by Gorgoulis et al. (36), which recommend that at least three independent senescence markers—combining cell-cycle arrest indicators, SA-*β*-galactosidase activity, and SASP factor expression—be assessed when characterizing senescent cells in tissues.

A related challenge concerns causality. Most human studies rely on placentas obtained at delivery, making it difficult to know whether observed senescence-like states actively drove disease progression or reflect the cumulative endpoint of a placenta exposed to prolonged injury. This uncertainty does not negate the model, but longitudinal and mechanistically integrated studies will be essential to move placental senescence from an interpretive framework to a clinically actionable biological target.

## 13 Conclusion

Premature placental senescence provides a mechanistically coherent framework for organizing the transition from abnormal placentation to the maternal syndrome in EOPE, supported by convergent data from histology, transcriptomics, secretome characterization, and circulating biomarker studies (6, 7, 12, 13). The causal chain from maladaptive placentation through syncytiotrophoblast senescence and SASP-mediated signal export to maternal endothelial injury has not been demonstrated longitudinally in humans. Alternative frameworks—decidual NK cell-mediated immune regulation, complement activation, and the sFlt-1/PlGF angiogenic axis—coexist with the senescence model (18–23). The operational definition of placental senescence requires refinement through consensus criteria, and the translational promise of senotherapeutics must be tempered by recognition that senescence serves physiological functions nonselective suppression could disrupt (18, 36). The most productive way forward is to treat the senescence hypothesis as a stimulus for more precise experimental design: identifying which trophoblast populations are most consequential, defining circulating signatures that most faithfully report that transition, and determining whether interventions improving trophoblast stress resolution can alter disease trajectory.

In practical terms, this framework narrows attention to testable transitions: when trophoblast stress ceases to be adaptive, how secretory reprogramming becomes stabilized, and which exported placental signals most faithfully indicate that disease has crossed from local dysfunction into systemic risk. Answering these questions will require longitudinal sampling, carefully phenotyped EOPE cohorts, integration of single-cell and spatial transcriptomic data with conventional histopathology, and functional validation in organoid or animal models.

Premature placental senescence should be regarded as a serious mechanistic candidate capable of integrating a fragmented body of literature on EOPE. The framework carries testable predictions: senescence markers should be detectable earlier in women who subsequently develop EOPE; circulating SASP-related factors should reflect the severity and timing of placental stress; and interventions improving trophoblast stress resolution should attenuate SASP output before affecting clinical endpoints. If future work—incorporating longitudinal cohorts, organoid-based mechanistic studies, single-cell and spatial transcriptomic resolution, and carefully designed intervention trials—can demonstrate when senescence begins and that modifying these processes alters outcomes, the concept will move from an interpretive model toward a clinically actionable mechanism.

## 14 Public health and pediatric implications

Although this review focused on molecular mechanisms linking premature placental senescence to the maternal syndrome in EOPE, the public health dimensions merit explicit consideration. As noted in the Introduction, preeclampsia accounts for an estimated 50,000–60,000 maternal deaths annually, predominantly in low- and middle-income countries (1). EOPE represents approximately 20%–25% of PE cases yet contributes disproportionately to severe maternal morbidity, indicated preterm delivery, and perinatal mortality. The public health burden is thus concentrated in the clinical phenotype for which the placental senescence framework is most mechanistically informative.

From a pediatric perspective, consequences of EOPE extend beyond the perinatal period. Infants born to preeclamptic pregnancies, particularly those delivered preterm or with fetal growth restriction (FGR, co-occurring in 30%–50% of EOPE), face elevated risks of neonatal complications, neurodevelopmental impairment, and increased lifetime risks of hypertension, obesity, type 2 diabetes, and cardiovascular disease (1, 2). A senescence-centered framework may clarify these associations by linking placental stress severity and timing to fetal nutrient and oxygen restriction, although direct evidence connecting specific SASP components to fetal programming remains limited (2, 17).

From a health systems perspective, first-trimester screening programs can identify women at high risk of preterm preeclampsia with detection rates of ~75% at a 10% screen-positive rate (36, 41). Adding senescence-linked biomarkers could improve risk stratification but requires analytical standardization, prospective validation, and cost-effectiveness analysis before implementation, particularly in low-resource settings where disease burden is highest (24, 25). Low-dose aspirin (150 mg/day) initiated before 16 weeks reduces preterm preeclampsia in high-risk women (37); the senescence model provides a biological rationale: by reducing platelet-derived inflammatory mediators, aspirin may attenuate the amplification stage of disease. Future research should evaluate whether senescence biomarkers can identify aspirin-responsive subgroups more precisely than current risk algorithms.
